# The relationship between muscle mass and fat content in body composition and non-alcoholic fatty liver disease in the Chinese general population: a cross-sectional study

**DOI:** 10.3389/fmed.2024.1384366

**Published:** 2024-06-10

**Authors:** Guoqiong Xu, Yuanyuan Wu, Jie Chen, Dan Xiang, Dongji Li

**Affiliations:** ^1^Health Medicine Center, The Second Affiliated Hospital of Chongqing Medical University, Chongqing Medical University, Chongqing, China; ^2^Department of Nursing, Armed Police Hospital of Chongqing, Chongqing, China; ^3^Department of Geriatrics, Armed Police Hospital of Chongqing, Chongqing, China

**Keywords:** non-alcocholic fatty liver disease, body composition, muscle mass, fat mass, liver function

## Abstract

**Introduction:**

Non-alcoholic fatty liver disease (NAFLD) poses a significant global health challenge, necessitating comprehensive exploration of its etiology. This study investigates the intricate relationship between body composition and NAFLD prevalence, focusing on the balance between muscle mass and fat content.

**Methods:**

Employing a retrospective cross-sectional design, 2,493 participants undergoing routine health examinations were analyzed. Body compositions, including muscle mass and fat, were measured using bioelectrical-impedance analysis. The prevalence of NAFLD was assessed based on clinical guidelines.

**Results:**

This study included 2,493 patients, including 1,601 (64.2%) men and 892(35.8%) women. The average age of these participants was 46.0 ± 13.1 years, with a mean body mass index of 25.0 ± 3.6 kg/m^2^. The levels of fat free mass (FFM) to fat mass (FM) ratio (FFM/FM) and appendicular skeletal muscle mass index (ASMI) demonstrated a negative association with the prevalence of NAFLD (OR (95% CI): 0.553 (0.427–0.704) and 0.850 (0.730–0.964), *p* < 0.001 and *p* = 0.022, respectively). Liver function further elucidates the multifaceted impact of body composition on hepatic health. In contrast to other parameters, FFM/FM displayed a negative association with liver damage indicators, including a negative association with alanine aminotransferase (Beta±SE: −1.00 ± 0.17, *p* < 0.001), with aspartate aminotransferase showing borderline significance (Beta±SE: −0.26 ± 0.15, *p* = 0.084). Similar associations were also evident in terms of liver productive function and bilirubin metabolism.

**Conclusion:**

Our study offers novel insights into the nuanced interplay between body composition and NAFLD. Recognizing the significance of the balance between muscle and fat provides a foundation for tailored interventions that may reshape the landscape of NAFLD prevention and management.

## Introduction

Non-alcoholic fatty liver disease (NAFLD) stands as a critical global public health issue, encompassing a spectrum of liver disorders characterized by the accumulation of excessive fat in the absence of alcohol abuse (1, 2). The contemporary surge in obesity and metabolic diseases, driven by pervasive changes in lifestyle, has propelled NAFLD to the forefront, establishing itself as the most prevalent liver condition globally. The World Health Organization reports that over 130 million individuals worldwide are affected by this condition, and this number is growing rapidly (3).

Despite its increasing prevalence and clinical significance, NAFLD management remains poorly understood, with no FDA-approved medications currently available (4). However, existing guidelines emphasize a healthy diet and regular exercise as fundamental pillars of NAFLD management (4). Notably, these lifestyle interventions are closely intertwined with body composition, underscoring the importance of understanding the relationship between body composition and NAFLD management.

In recent years, an increasing body of evidence has shed light on the intricate interplay between body compositions and NAFLD (5–7). Researchers have recognized that certain components of body composition, including fat distribution, adipose tissue characteristics, as well as muscle mass, play significant roles in the development and progression of NAFLD. For instance, a high visceral fat area (VFA) and low appendicular skeletal muscle mass (ASM) have been linked to NAFLD progression (8–10). Indeed, visceral fat accumulation has been strongly associated with insulin resistance, dyslipidemia, and inflammation, all of which are key contributors to NAFLD pathogenesis. Meanwhile body composition improvement was suggested to be associated with steatosis resolution in NAFLD (11). However, a substantial knowledge gap persists concerning the nuanced association between body compositions, especially muscle mass, and NAFLD among the general population. The current state of research reveals a multifaceted relationship between body compositions and NAFLD. While high VFA has been consistently associated with an increased risk of NAFLD, the role of low ASM in this context is less understood. Existing studies often focus on individual components rather than considering the delicate balance between muscle mass and fat content.

Our research is motivated by the aspiration to elucidate whether heightened body compositions correlate with the prevalence of NAFLD and to gain deeper insights into the potential role of body compositions in maintaining hepatic health. Through this investigation, our aim is to provide a novel perspective and propose potential interventions for the prevention and treatment of NAFLD. The central objective of this study is to scrutinize the relationship between body compositions, especially the balance between muscle mass and fat, and the prevalence of NAFLD within the Chinese population undergoing routine health examinations. Our hypothesis posits that individuals with elevated muscle mass and lower fat levels exhibit a lower risk of NAFLD compared to those with lower muscle mass but higher fat levels. In addition, we explore the relationship between body compositions and liver function, aiming to uncover the intricate physiological mechanisms that contribute to sustaining liver health. This study is poised to furnish a robust scientific foundation for the development of more effective strategies for the intervention of NAFLD, taking into account the delicate interplay between muscle mass and fat content in the broader context of body compositions.

## Methods

### Study design and population

This retrospective cross-sectional study utilized historical medical records from individuals undergoing health screenings at the Department of Health Management Center of the Second Affiliated Hospital of Chongqing Medical University in Chongqing, China between June 2017 and January 2023. The study received approval from the Ethics Committee of the Second Affiliated Hospital of Chongqing Medical University and adhered to the principles of the Helsinki Declaration. To protect patient privacy, data were collected by clinic ID number rather than name, and informed consent was waived.

We initially recruited 2,667 participants who underwent body compositions examination during 2016–2023. Our trained nurse collected information on demographic characteristics (smoking, alcohol consumption, and physical exercise) and medical history (hypertension, diabetes, and viral hepatitis) through a standard questionnaire. Medical records from January 2016 to August 2022 were retrieved from the hospital information system (HIS), and participants were selected for this study according to the following inclusion criteria: (1) they were aged ≥18 years; (2) they had completed the questionnaire with information of demographic characteristics and health-related habits being available; (3) they had body compositions examination; and (4) they had medical tests for liver function, kidney function and blood lipid profile. Exclusion criteria included: (1) subjects with missing data were excluded in data analysis (*N* = 42); (2) subjects with alcohol consumption ≥ 140 g/week for men or ≥ 70 g/week for women (*N* = 38); (3) subjects with extreme low muscle mass (*N* = 22) and body mass index (BMI) (*N* = 18); (4) We further excluded the following patients by reviewing their electronic medical records; laboratory data and test results more than 1 month apart (*n* = 54). Finally, 2,493 patients were included in this study (Supplementary Figure S1).

### Definition of outcomes

NAFLD diagnosis was based on the clinical practice guidelines for NAFLD management in the Asia-Pacific and the Chinese Medical Association’s Hepatology Branch (12, 13). The diagnostic criteria were as follows: (1) hepatic steatosis identified by color Doppler ultrasound; (2) no history of drinking alcohol or alcohol intake in an equivalent amount (< 140 g/week for men and < 70 g/week for women); (3) exclusion of subjects with diseases that could cause fatty liver, such as viral hepatitis, autoimmune liver disease, and drug-induced liver disease.

Liver functions were categorized into three group: productive function [total protein (TP), albumin (ALB), globulin (GLOB), cholinesterase (CHE), pre-albumin (PAB)], liver damages [alanine aminotransferase (ALT), aspartate aminotransferase (AST)], and bilirubin metabolism [total bilirubin (TBIL), direct bilirubin (DBIL), γγ—glutamyl transferase (GGT), alkaline phosphatase (AKP)]. The test was conducted using a Hitachi automatic biochemistry analyzer in our hospital’s central laboratory, which has passed external quality assessment and capability certification. The instruments underwent strict quality control testing prior to use.

### Body composition measurement

On the same day as the ultrasound test, body composition data was measured using a bioelectrical-impedance body composition analyzer (BIA) (Qinghua Tongfang BCA-2A, Beijing, China), and technicians were blinded to the clinical information. Height, weight, waist circumference (WC), BMI, percent body fat (PBF), VFA, ASM, appendicular skeletal muscle mass index (ASMI), fat mass (FM), fat free mass (FFM), FFM to FM ratio (FFM/FM) were the body data examined in this study. We also measured the fat and muscle in different areas, including fat in right leg, fat in left leg, fat in right arm, fat in left arm, fat in trunk, muscle in right leg, muscle in left leg, muscle in right arm, muscle in left arm, and muscle in trunk. ASMI was corrected for height (ASMI = ASM (kg)/height (m^2^), kg/m^2^). According to the Asian Working Group for Sarcopenia (AWGS) criteria, sarcopenia is defined as ASMI < 7.0 kg/ m^2^ in men and < 5.7 kg/ m^2^ in women (measured by BIA) (14). Visceral obesity was defined as VFA ≥ 100 cm^2^ in both sexes (15).

### Measurements of covariates

Covariates were selected based on our previous study and other studies, as well as risk factors for NAFLD (14, 15). Smoking was categorized as smoker and nonsmoker. Alcohol consumption is classified as follows: non-drinker (0 g/week), light drinker (< 140 g/week for men and < 70 g/week for women), and heavy drinker (≥ 140 g/week for men and ≥ 70 g/week for women). Physical exercise was stratified as follows: low (no exercise), moderate (3 days/week or less), high (3 days/week or more) (physical activity is defined as at least 30–60 min/day of moderate-intensity exercise). Body weight and height were measured by trained nurses following the standard procedures, and BMI was calculated as weight (kg) divided by height (m) squared. Systolic blood pressure (SBP) and diastolic blood pressure (DBP) were measured automatically by using an Omron digital monitor. Venous blood was collected in the morning after an overnight fasting for 8–10 h, and all blood samples were used for biochemical tests following the standard laboratory procedures. These tests included, but not limited to, fasting glucose (FBG, mmol/L), Hemoglobin (HbA1c, %), triglyceride (TG, mmol/L), total cholesterol (TC, mmol/L), low-density lipoprotein cholesterol (LDL-c, mmol/L), high-density lipoprotein cholesterol (HDL-c, mmol/L), urine acid (UA, μmol/L).

Hypertension (HBP) was defined as SBP ≥ 140 mmhg or DBP ≥ 90 mmhg or diagnosed by doctor before. Type 2 diabetes mellitus (T2DM) was defined as FBG ≥ 7.0 mmol/L or 2-h plasma glucose ≥ 11.1 mmol/L or HbA1c ≥ 6.5%, and/or treatment with anti-diabetic medication currently or diagnosed by doctor before (16). The definition of dyslipidemia was TG ≥ 2.3 mmol/L or TC ≥ 6.2 mmol/L or HDL < 1.0 mmol/L or LDL ≥ 4.1 mmol/L, and/or currently taking antihyperlipidemic medication or diagnosed by doctor before (17).

### Statistical analyses

Data are expressed as mean ± SD, median (Q1, Q3), or proportion (%) as appropriate. We examined the associations between body compositions and the prevalence of NAFLD using logistic regression and the association between body compositions and liver function using linear regression. Covariates included age, sex, BMI, smoking status, drinking status, FBG, TG, TC, physical activity and the status of diabetes. Dose–response curves for associations between body compositions and NAFLD were assessed through restricted cubic spline regression models. Participants with missing covariate values were excluded from the study. We compared the characteristics between patients with and without missing data. We repeated analyses stratified by age, sex, BMI, smoking status, drinking status and diabetes as sensitivity analysis. We also tested the interaction effect between body compositions and age, sex, smoking status, drinking status and diabetes on prevalent NAFLD. The analysis was conducted using the R program (version 4.0) for data cleaning and analyses. A two-sided *p*-value of <0.05 was the threshold for statistical significance.

## Results

### Participants’ characteristics

This study included 2,493 patients, including 1,601 (64.2%) men and 892 (35.8%) women. The average age of these participants was 46.0 ± 13.1 years, with a mean BMI of 25.0 ± 3.6 kg/m^2^. Among them, 303 (12.2%) were smokers and 485 (19.5%) were drinkers. Table 1 summarizes the clinical characteristics of patients stratified status of NAFLD. Compared with subjects without NAFLD, subjects with NAFLD were older (49.1 ± 10.5 vs. 45.0 ± 13.9, *p* < 0.001), had a higher proportion of males (85.1% vs. 55.1%, *p* < 0.001), as well as a higher level of BMI (26.7 ± 3.0 vs. 24.0 ± 3.5, *p* < 0.001) and WHR (0.92 ± 0.06 vs. 0.85 ± 0.07, *p* < 0.001). Regarding comorbidities, subjects with NAFLD also exhibited a higher prevalence of diabetes (11.9% vs. 3.8%, *p* < 0.001), hypertension (19.1% vs. 4.5%, *p* < 0.001) and dyslipidemia (4.0% vs.0.6%, *p* < 0.001).

**Table 1:** The comparison of clinical characteristics between subjects with and without NAFLD.

Variables	Overall	Subjects without NAFLD	Subjects with NAFLD	*p* value
Number	2,493	1734	759	
Age [mean (SD)]	46.0 (13.1)	45.0 (13.9)	49.1 (10.5)	<0.001
Male [*N*(%)]	1,601 (64.2)	955 (55.1)	646 (85.1)	<0.001
Smoker [*N*(%)]	303 (12.2)	149 (8.6)	154 (20.3)	<0.001
Drinker [*N*(%)]	485 (19.5)	235 (13.6)	250 (32.9)	<0.001
Physical activity [*N*(%)]				
Low	383 (34.6)	209 (32.6)	174 (37.4)	0.239
Moderate	388 (35.1)	234 (36.5)	154 (33.1)	
High	335 (30.3)	198 (30.9)	137 (29.5)	
BMI [mean (SD)]	25.0 (3.6)	24.0 (3.5)	26.7 (3.0)	<0.001
WHR [mean (SD)]	0.90 (0.07)	0.85 (0.07)	0.92 (0.06)	<0.001
SBP [mean (SD)]	120 (17)	121 (17)	128 (17)	<0.001
DBP [mean (SD)]	75 (12)	73 (11)	79 (12)	<0.001
TP [mean (SD)]	78.0 (5.5)	77.4 (5.3)	78.1 (5.8)	0.008
ALB [mean (SD)]	46.0 (2.8)	45.7 (2.9)	46.2 (2.8)	<0.001
GLOB [mean (SD)]	32.0 (5.0)	31.6 (4.8)	31.9 (5.4)	0.207
CHE [mean (SD)]	9.4 (2.0)	9.2 (2.0)	10.3 (1.8)	<0.001
PAB [mean (SD)]	290.0 (57.2)	280.6 (55.4)	310.2 (58.3)	<0.001
ALT [mean (SD)]	27.0 (22.5)	23.5 (19.7)	35.6 (25.7)	<0.001
AST [mean (SD)]	24.0 (19.8)	22.6 (22.4)	26.0 (12.1)	<0.001
TBIL [mean (SD)]	12.0 (5.5)	11.2 (5.5)	12.5 (5.5)	<0.001
DBIL [mean (SD)]	3.5 (2.5)	3.5 (2.8)	3.5 (1.8)	0.827
GGT [mean (SD)]	38.0 (39.9)	30.5 (31.2)	53.3 (49.7)	<0.001
AKP [mean (SD)]	71.0 (26.6)	70.6 (21.1)	74.7 (41.4)	0.077
Diabetes [*N*(%)]	156 (6.3)	66 (3.8)	90 (11.9)	<0.001
Hypertension [*N*(%)]	223 (8.9)	78 (4.5)	145 (19.1)	<0.001
CHD [*N*(%)]	25 (1.0)	8 (0.5)	17 (2.2)	<0.001
Hyperlipidemia [*N*(%)]	40 (1.6)	10 (0.6)	30 (4.0)	<0.001

NAFLD, Non-alcoholic fatty liver disease; BMI, body mass index; WHR, waist hip ratio; SBP, Systolic blood pressure; DBP, diastolic blood pressure; TP, total protein; ALB, albumin; GLOB, globulin; CHE, cholinesterase; PAB, pre-albumin; ALT, alanine aminotransferase; AST, aspartate aminotransferase; TBIL, total bilirubin; DBIL, direct bilirubin; GGT, γ—glutamyl transferase; AKP, alkaline phosphatase; CHD, coronary heart disease.

### The relationship between body compositions and prevalent NAFLD

The analysis revealed compelling associations between various body compositions and the prevalence of NAFLD. After meticulous adjustment for covariates, elevated levels of PBF and VFA were notably linked to a heightened risk of prevalent NAFLD [OR (95% CI): 1.080 (1.036–1.129) and 1.280 (1.113–1.479), both *p* value <0.001, Table 2]. Additionally, FM demonstrated a similar association with NAFLD, albeit at borderline significance [OR (95% CI): 1.054 (1.000–1.113), *p* = 0.050]. Conversely, the levels of FFM/FM and ASMI demonstrated a negative association with the prevalence of NAFLD, and this association remained independent of common confounders [OR (95% CI): 0.553 (0.427–0.704) and 0.850 (0.730–0.964), *p* < 0.001 and *p* = 0.022, Table 2]. Notably, although the odds ratios of ASM and FFM showed a consistent direction, they did not achieve statistical significance following full adjustment. Moreover, upon additional adjustment for physical activity (model 4 in Supplementary Table S1), these significant associations persisted. Both ASMI and FFM/FM maintained a negative association with NAFLD [OR (95% CI): 0.608 (0.417–0.863) and 0.742 (0.553–0.955), *p* = 0.007 and 0.031, respectively], while PBF, VFA, and FM remained positively associated with NAFLD [OR (95% CI): 1.112 (1.043–1.194), 1.468 (1.205–1.822), and 1.220 (1.110–1.351), *p* = 0.002, < 0.0001, and < 0.0001, respectively]. This section of results was relegated to the Supplementary material rather than the main results due to the significant proportion (56.2%) of missing values in the recordings of physical activity.

**Table 2:** The relationship between body compositions and NAFLD.

Variable	Model 0		Model 1		Model 2		Model 3	
	OR (95%CI)	*p* value	OR (95%CI)	*p* value	OR (95%CI)	*p* value	OR (95%CI)	*p* value
Water	1.107 (1.092–1.123)	<0.001	1.110 (1.088–1.133)	<0.001	1.017 (0.990–1.044)	0.217	0.999 (0.969–1.029)	0.941
Bone mass	4.074 (3.377–4.936)	<0.001	4.206 (3.177–5.599)	<0.001	1.260 (0.878–1.812)	0.211	0.988 (0.654–1.494)	0.955
Protein mass	1.435 (1.367–1.508)	<0.001	1.445 (1.344–1.556)	<0.001	1.058 (0.964–1.162)	0.233	0.993 (0.893–1.105)	0.903
Muscle mass	1.083 (1.071–1.095)	<0.001	1.085 (1.068–1.103)	<0.001	1.013 (0.993–1.034)	0.213	0.999 (0.976–1.023)	0.949
PBF	1.025 (1.011–1.039)	0.001	1.163 (1.137–1.190)	<0.001	1.062 (1.025–1.102)	0.001	1.080 (1.036–1.129)	<0.001
FM	1.100 (1.082–1.118)	<0.001	1.138 (1.118–1.160)	<0.001	1.059 (1.010–1.111)	0.018	1.054 (1.000–1.113)	0.05
FFM	1.078 (1.067–1.089)	<0.001	1.080 (1.064–1.097)	<0.001	1.012 (0.993–1.032)	0.213	0.999 (0.977–1.022)	0.946
FFM/FM	0.754 (0.684–0.828)	<0.001	0.362 (0.308–0.424)	<0.001	0.577 (0.464–0.710)	<0.001	0.553 (0.427–0.704)	<0.001
ASM	1.086 (1.071–1.101)	<0.001	1.053 (1.034–1.074)	<0.001	1.001 (0.981–1.019)	0.902	0.984 (0.958–1.009)	0.229
ASMI	1.408 (1.327–1.496)	<0.001	1.203 (1.125–1.294)	<0.001	0.942 (0.845–1.020)	0.214	0.850 (0.730–0.964)	0.022
BMR	1.002 (1.001–1.002)	<0.001	1.003 (1.002–1.004)	<0.001	1.001 (1.000–1.002)	0.05	1.000 (0.999–1.001)	0.713
VFA	1.516 (1.445–1.594)	<0.001	1.571 (1.491–1.657)	<0.001	1.359 (1.203–1.541)	<0.001	1.280 (1.113–1.479)	0.001

NAFLD, Non-alcoholic fatty liver disease; PBF, percent body fat, FM, fat mass; FFM, fat free mass; FFM/FM, FFM to FM ratio; ASM, appendicular skeletal muscle mass; ASMI, appendicular skeletal muscle mass index; BMR, basal metabolic rate; VFA, visceral fat area.

### The relationship between body composition and liver function

Next, we explored the relationship between body compositions and liver function across three dimensions: productive function, liver damages, and bilirubin metabolism. In terms of liver productive function, as shown in Table 3, several parameters related to fat and muscle (namely, PBF, FM, VFA and FFM/FM) exhibited significant associations with total protein, with these associations primarily driven by globulin rather than albumin. Notably, opposite to the other body composition parameters, FFM/FM displayed a significant negative association with both total protein and globulin (Beta ± SE: −0.11 ± 0.04 and − 0.12 ± 0.04, *p* = 0.013 and 0.003, respectively). Similar patterns were observed for cholinesterase and pre-albumin, two other indicators of liver productive function (Beta ± SE: −0.45 ± 0.05 and − 2.76 ± 1.56, *p* < 0.001 and = 0.077, respectively; see Supplementary Table S2). Similar associations were also evident in terms of liver damage and bilirubin metabolism (refer to Table 2 and Supplementary Table S2). In contrast to other parameters, FFM/FM demonstrated a negative association with ALT (Beta ± SE: −1.00 ± 0.17, *p* < 0.001), with AST showing borderline significance (Beta ± SE: −0.26 ± 0.15, *p* = 0.084), as well as with rGT and ALP (Beta ± SE: −1.56 ± 0.41 and − 2.08 ± 0.77, *p* < 0.001 and = 0.007, respectively).

**Table 3:** The relationship between body compositions and liver functions.

Variables	Protein production						Liver damages			
	TP		ALB		GLOB		ALT		AST	
	Beta ± Se	*p* value	Beta ± Se	*p* value	Beta ± Se	*p* value	Beta ± Se	*p* value	Beta ± Se	*p* value
WATER	0.05 ± 0.03	0.067	−0.01 ± 0.01	0.589	0.06 ± 0.02	0.019	1.09 ± 0.10	<0.001	0.31 ± 0.09	0.001
Bone mass	0.65 ± 0.35	0.068	−0.09 ± 0.17	0.59	0.78 ± 0.33	0.019	15.03 ± 1.32	<0.001	4.21 ± 1.24	0.001
Protein mass	0.17 ± 0.09	0.07	−0.02 ± 0.04	0.578	0.20 ± 0.09	0.019	3.88 ± 0.34	<0.001	1.08 ± 0.32	0.001
Muscle mass	0.04 ± 0.02	0.067	−0.01 ± 0.01	0.588	0.04 ± 0.02	0.019	0.85 ± 0.07	<0.001	0.24 ± 0.07	0.001
PBF	0.13 ± 0.02	<0.001	−0.01 ± 0.01	0.347	0.15 ± 0.02	<0.001	1.27 ± 0.09	<0.001	0.44 ± 0.08	<0.001
FM	0.11 ± 0.02	<0.001	−0.01 ± 0.01	0.181	0.12 ± 0.02	<0.001	1.28 ± 0.07	<0.001	0.44 ± 0.07	<0.001
FFM	0.03 ± 0.02	0.069	−0.01 ± 0.01	0.584	0.04 ± 0.02	0.019	0.81 ± 0.07	<0.001	0.23 ± 0.07	0.001
FFM/FM	−0.11 ± 0.04	0.013	0.01 ± 0.02	0.607	−0.12 ± 0.04	0.003	−1.00 ± 0.17	<0.001	−0.26 ± 0.15	0.084
ASM	0.02 ± 0.02	0.213	−0.01 ± 0.01	0.548	0.03 ± 0.02	0.088	0.48 ± 0.07	<0.001	0.13 ± 0.06	0.046
ASMI	0.08 ± 0.06	0.176	−0.03 ± 0.03	0.279	0.12 ± 0.06	0.035	1.51 ± 0.22	<0.001	0.47 ± 0.20	0.02
BMR	0.00 ± 0.00	0.004	0.00 ± 0.00	0.795	0.00 ± 0.00	0.002	0.04 ± 0.00	<0.001	0.01 ± 0.00	<0.001
VFA	0.22 ± 0.05	<0.001	−0.05 ± 0.02	0.047	0.29 ± 0.05	<0.001	2.67 ± 0.17	<0.001	0.82 ± 0.19	<0.001

PBF, percent body fat, FM, fat mass; FFM, fat free mass; FFM/FM, FFM to FM ratio; ASM, appendicular skeletal muscle mass; ASMI, appendicular skeletal muscle mass index; BMR, basal metabolic rate; VFA, visceral fat area; TP, total protein; ALB, albumin; GLOB, globulin; ALT, alanine aminotransferase; AST, aspartate aminotransferase.

### Sensitivity analysis

**Figure 1:**
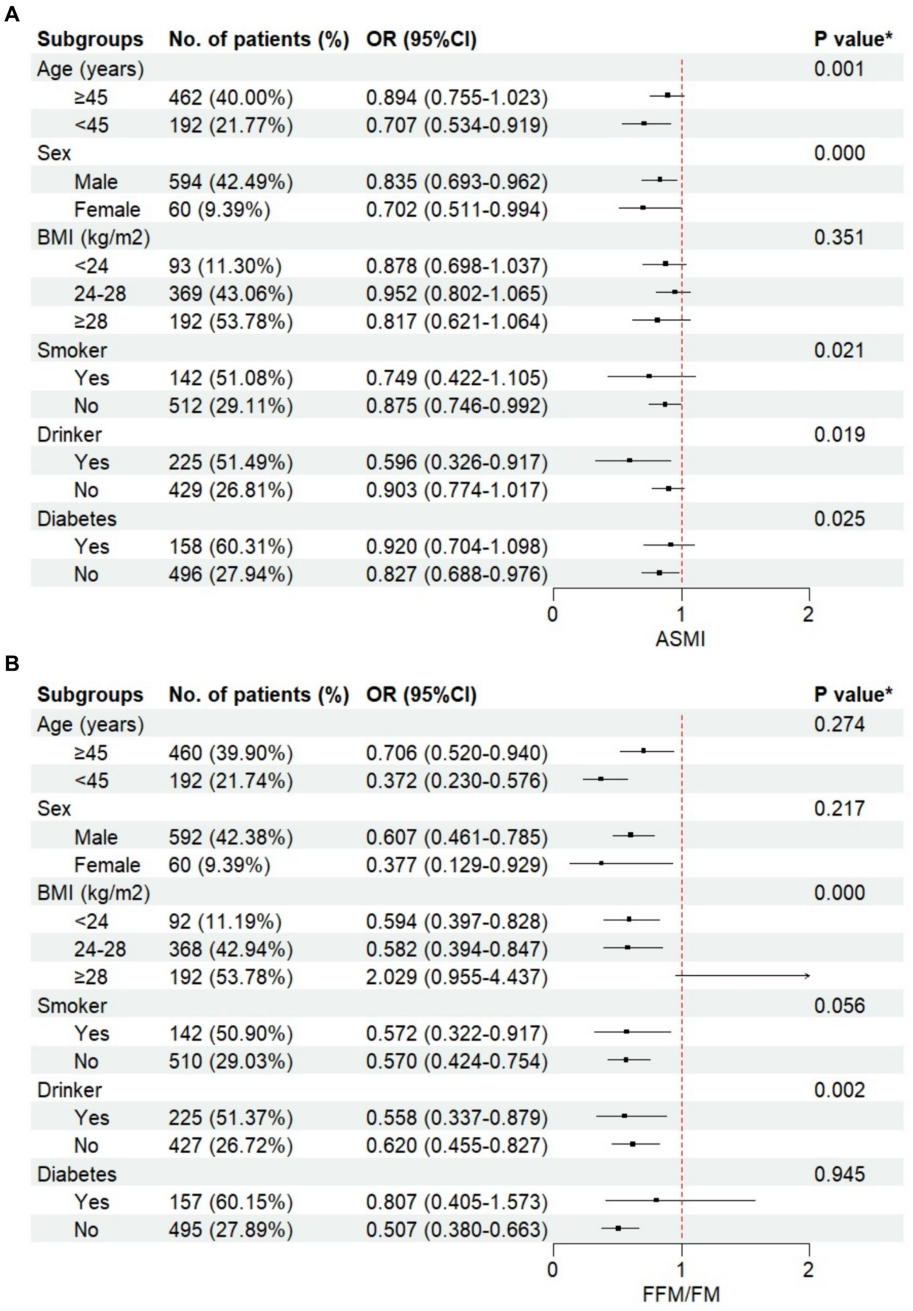
The relationship between body compositions and non-alcoholic fatty liver disease (NAFLD) stratified by different groups. **(A)** appendicular skeletal muscle mass index (ASMI), **(B)** fat free mass to fat mass ratio (FFM/FM).

As depicted in Figure 1, when stratified by age, sex, BMI, smoking, drinking, and diabetes, FFM/FM exhibited a consistent negative association with prevalent NAFLD, except for subjects with a BMI ≥ 28 kg/m^2^ [OR (95% CI): 2.029 (0.955–4.437)], but this association did not reach statistical significance. Significant interaction effects were observed between FFM/FM and BMI/drinking status on prevalent NAFLD (P for interaction <0.001 and = 0.002, respectively). Similarly, ASMI consistently showed a negative association with prevalent NAFLD within each subgroup. The interaction effect between ASMI and sex/smoking/drinking/diabetes on prevalent NAFLD was also significant (P for interaction <0.001, = 0.021, 0.019 and 0.025, respectively).

## Discussion

NAFLD has emerged as a major global public health concern, and understanding its intricate relationship with body composition is crucial for developing optimal management. This study is aimed at investigating the significance of the balance between muscle and fat mass concerning NAFLD risk. After a comprehensive analysis of the associations between various body compositions and the prevalence of NAFLD, our findings demonstrated that individuals with heightened muscle mass and lower fat levels was associated with a decreased risk of NAFLD. Moreover, a balanced ratio of muscle to fat mass was associated with favorable changes of liver health.

Our findings were in line with previous studies that higher levels of body fat, as indicated by increase in PBF, FM, and VFA, were robustly associated with a higher risk of NAFLD (8–11, 18). More importantly, our study unveils a relatively less explored aspect: the significance of not only fat mass but also muscle mass, particularly their balance, in NAFLD management. We observed that individuals with elevated ASMI and FFM/FM exhibited a decreased risk of NAFLD, underscoring the importance of considering both muscle and fat mass in NAFLD health management. Apart from the importance of fat mass, researchers recently begin to recognize that muscle mass also plays a crucial role in NAFLD pathogenesis. As Cai et al. (18) reported, the presence and severity of hepatic steatosis is negatively affected by not only increased visceral or subcutaneous adipose tissue, but also decreased skeletal or appendicular muscle mass. They found patients with NAFLD had lower levels of muscle mass than healthy subjects, and patients with sarcopenia had higher risk of having NAFLD, as well as NASH or NAFLD-related significant fibrosis (18). Moreover, in a meta-analysis of longitudinal studies, Mátis et al. reported that muscle mass increase was positively associated with liver steatosis decrease and NAFLD resolution (11, 16, 19). Thus, our results are in accordance with others that both muscle and fat mass are of vital importance for NAFLD management.

Besides, the relationship between body composition and liver function further supported the impact of body composition on hepatic health. Specifically, in contrast to other fat- or muscle-related parameters (e.g., PBF, FM, VFA, FFM, ASM, or ASMI), FFM/FM demonstrated a negative association with both ALT and AST that representing liver damage. Similar patterns were also observed for liver productive function and bilirubin metabolism, where FFM/FM was inversely associated with total protein, globulin, rGT, as well as ALP. These findings further emphasized the importance of the balance between muscle and fat mass in mitigating NAFLD risk.

Furthermore, sensitivity analyses strengthened the robustness of our results, demonstrating the consistency of associations across diverse demographic and health-related subgroups. Notably, FFM/FM maintained a negative association with prevalent NAFLD across various strata, indicating its potential as a universal indicator. The interaction analyses also highlighted the nuanced impact of BMI and drinking status on the association between FFM/FM and NAFLD. The positive associations between ASMI and NAFLD in all groups further emphasize the protective role of muscle mass in hepatic health. These findings align with emerging evidence suggesting that maintaining optimal muscle mass may be a crucial factor in preventing metabolic disorders such as NAFLD. The significant interaction analyses with sex, smoking, drinking, and diabetes underscore the importance of considering individual characteristics in understanding the complex interplay between body composition and NAFLD. Incorporating physical activity into the analysis revealed additional insights. Both ASMI and FFM/FM remained significantly associated with NAFLD, underscoring the importance of muscle mass and its ratio to fat mass in influencing liver health.

NAFLD is intricately linked to the delicate balance between muscle and fat content within the body. It is widely acknowledged that weight loss and modification of body composition can contribute to improving NAFLD and preventing life-threatening diseases (17, 19). Weight loss usually involves reductions in both body fat and muscle mass, whereas low muscle mass (with or without high body fat mass), such as patients with sarcopenia, often exhibit liver steatosis and fibrosis (11, 18). Thus, it is plausible that not the body weight reduction, but the reduction of body fat and the increase in skeletal muscle mass is crucial for NAFLD (19). Our study, exploring the association between body composition and NAFLD, unraveled a complex interplay that extends beyond traditional adiposity markers. One pivotal aspect of this interplay is the equilibrium between muscle and fat content, a balance that proved to have significant implications for hepatic health. The balance between muscle mass and fat content emerged as a crucial determinant of NAFLD prevalence. Our findings consistently highlighted that individuals with elevated muscle mass and a lower fat level exhibited a reduced risk of NAFLD. This equilibrium suggests a protective role of muscle tissue against the development of hepatic steatosis (20, 21).

The potential mechanisms underlying this protective effect warrant exploration to unveil the intricate biology governing the relationship between muscle and liver health. One plausible mechanism is the role of skeletal muscle in energy metabolism (22, 23). Skeletal muscles act as a metabolic powerhouse, playing a central role in glucose and lipid homeostasis. Increased muscle mass enhances insulin sensitivity, leading to improved glucose utilization and reduced lipogenesis (24–26). This, in turn, could mitigate the excessive accumulation of fat within the liver, a hallmark of NAFLD. The balance between muscle and fat might contribute to a more efficient energy utilization pattern, thereby protecting the liver from lipid overload. Moreover, muscle tissue is metabolically active, and its presence could contribute to the secretion of myokines—bioactive molecules with potential anti-inflammatory and metabolic effects (27, 28). These myokines may exert systemic effects, influencing not only muscle function but also modulating hepatic metabolism. The intricate signaling network between muscle and liver tissue requires further exploration to decipher the specific molecules and pathways involved in mediating the protective effects observed in our study.

The clinical significance of understanding the balance between muscle and fat in the context of NAFLD extends beyond risk assessment. Interventions aimed at optimizing this balance could hold promise for the prevention and management of NAFLD. Physical activity and resistance training, known promoters of muscle mass, may emerge as valuable strategies to enhance this equilibrium and reduce the risk of hepatic steatosis (29, 30). Tailoring lifestyle interventions to focus on preserving or increasing muscle mass might represent a novel avenue for NAFLD management.

Our study prompts a paradigm shift in how we perceive the relationship between body composition, particularly the balance between muscle and fat, and NAFLD. Recognizing the dynamic interplay between these components opens avenues for personalized interventions and highlights the potential of muscle-centric approaches in the clinical management of NAFLD. Future research endeavors should delve deeper into the molecular mechanisms, therapeutic implications, and long-term outcomes associated with optimizing the delicate equilibrium between muscle and fat in the context of hepatic health.

Our study has several strengths, including a large sample size, comprehensive body composition measurements, and meticulous adjustment for confounding factors. However, certain limitations should be acknowledged. The cross-sectional design precludes establishing causation, and longitudinal studies are warranted to elucidate the temporal relationship between body composition changes and NAFLD development. Additionally, our study focused on a specific population undergoing routine health examinations, and generalizability to other populations should be approached with caution.

In conclusion, our study provides robust evidence on the associations between body compositions and the prevalence of NAFLD, underscoring the importance for targeted interventions that prioritize muscle-to-fat ratio maintenance to control NAFLD. The nuanced relationships highlighted the importance of considering diverse body composition parameters beyond traditional adiposity measures. These findings contribute to the growing body of literature emphasizing the role of muscle mass and its ratio to fat mass in influencing metabolic health. Future research should delve into the mechanisms underlying these associations and explore interventions targeting optimal body composition for preventing and managing NAFLD.

## Data availability statement

The raw data supporting the conclusions of this article will be made available by the corresponding author, without undue reservation.

## Ethics statement

The studies involving humans were approved by the Ethics Committee of the Second Affiliated Hospital of Chongqing Medical University. The studies were conducted in accordance with the local legislation and institutional requirements. The informed consent was waived.

## Author contributions

GX: Conceptualization, Data curation, Formal analysis, Investigation, Writing – original draft, Writing – review & editing. YW: Conceptualization, Data curation, Formal analysis, Investigation, Methodology, Resources, Writing – original draft, Writing – review & editing. JC: Writing – original draft, Writing – review & editing. DX: Writing – original draft, Writing – review & editing. DL: Conceptualization, Data curation, Formal analysis, Writing – original draft, Writing – review & editing, Investigation, Methodology, Project administration.
