# Supplementary material for: The relationship between muscle mass and fat content in body composition and non-alcoholic fatty liver disease in the Chinese general population: a cross-sectional study
Source: Front Med (Lausanne). 2024 Jun 10;11:1384366. doi: 10.3389/fmed.2024.1384366 (PMC11194319; doi:10.3389/fmed.2024.1384366)
Supplement: Supplementary file 1 [file Data_Sheet_1.docx]

Table S1 Sensitivity analysis by further adjusting for physical activity

| Variables | Model 4 | |
| --- | --- | --- |
|  | OR (95%CI) | P value |
| WATER | 0.999 (0.952-1.048) | 0.969 |
| PBF | 1.112 (1.043-1.194) | 0.002 |
| FM | 1.220 (1.110-1.351) | 0.000 |
| Bone mass | 0.987 (0.511-1.908) | 0.969 |
| Protein mass | 0.994 (0.839-1.178) | 0.944 |
| Muscle mass | 0.999 (0.962-1.037) | 0.959 |
| FFM | 0.999 (0.964-1.035) | 0.964 |
| FFM/FM | 0.608 (0.417-0.863) | 0.007 |
| ASM | 0.992 (0.946-1.039) | 0.747 |
| ASMI | 0.742 (0.553-0.955) | 0.031 |
| BMR | 1.001 (0.999-1.002) | 0.359 |
| VFA | 1.468 (1.205-1.822) | 0.000 |

Abbreviation: NAFLD, Non-alcoholic fatty liver disease; PBF, percent body fat, FM, fat mass; FFM, fat free mass; FFM/FM, FFM to FM ratio; ASM, appendicular skeletal muscle mass; ASMI, appendicular skeletal muscle mass index; BMR, basal metabolic rate; VFA, visceral fat area.

Table S2 The relationship between body compositions and liver functions.

| Variables | Protein production | | | |  | Bilirubin metabolism | | | | | | | |
| --- | --- | --- | --- | --- | --- | --- | --- | --- | --- | --- | --- | --- | --- |
|  | CHE | | PAB | |  | TBIL | | DBIL | | GGT | | AKP | |
|  | Beta ± Se | P value | Beta ± Se | P value |  | Beta ± Se | P value | Beta ± Se | P value | Beta ± Se | P value | Beta ± Se | P value |
| WATER | 0.11±0.01 | <0.001 | 1.10±0.38 | 0.003 |  | -0.01±0.03 | 0.788 | -0.02±0.01 | 0.096 | 1.29±0.20 | <0.001 | 0.06±0.19 | 0.757 |
| Bone mass | 1.54±0.17 | <0.001 | 15.11±5.18 | 0.004 |  | -0.09±0.36 | 0.797 | -0.33±0.20 | 0.099 | 17.71±2.74 | <0.001 | 0.73±2.57 | 0.776 |
| Protein mass | 0.40±0.04 | <0.001 | 3.91±1.34 | 0.004 |  | -0.03±0.09 | 0.744 | -0.09±0.05 | 0.092 | 4.58±0.71 | <0.001 | 0.18±0.66 | 0.786 |
| Muscle mass | 0.09±0.01 | <0.001 | 0.86±0.29 | 0.003 |  | -0.01±0.02 | 0.799 | -0.02±0.01 | 0.097 | 1.01±0.16 | <0.001 | 0.04±0.15 | 0.767 |
| PBF | 0.14±0.01 | <0.001 | 1.22±0.37 | 0.001 |  | -0.09±0.02 | <0.001 | -0.04±0.01 | 0.009 | 1.52±0.18 | <0.001 | 0.94±0.18 | <0.001 |
| FM | 0.12±0.01 | <0.001 | 1.00±0.29 | 0.001 |  | -0.07±0.02 | 0.001 | -0.04±0.01 | 0.004 | 1.51±0.16 | <0.001 | 0.64±0.14 | <0.001 |
| FFM | 0.08±0.01 | <0.001 | 0.82±0.28 | 0.003 |  | 0.00±0.02 | 0.8 | -0.02±0.01 | 0.098 | 0.95±0.15 | <0.001 | 0.04±0.14 | 0.768 |
| FFM/FM | -0.45±0.05 | <0.001 | -2.76±1.56 | 0.077 |  | 0.07±0.04 | 0.096 | 0.02±0.02 | 0.441 | -1.56±0.41 | <0.001 | -2.08±0.77 | 0.007 |
| ASM | 0.11±0.01 | <0.001 | 1.22±0.39 | 0.002 |  | 0.00±0.02 | 0.847 | -0.01±0.01 | 0.241 | 0.60±0.15 | <0.001 | 0.07±0.19 | 0.717 |
| ASMI | 0.49±0.05 | <0.001 | 5.38±1.46 | <0.001 |  | -0.06±0.06 | 0.292 | -0.03±0.03 | 0.247 | 1.88±0.47 | <0.001 | 2.04±0.72 | 0.005 |
| BMR | 0.00±0.00 | <0.001 | 0.03±0.01 | 0.009 |  | 0.00±0.00 | 0.639 | 0.00±0.00 | 0.054 | 0.04±0.01 | <0.001 | 0.00±0.01 | 0.635 |
| VFA | 0.24±0.03 | <0.001 | 2.09±0.97 | 0.032 |  | -0.08±0.06 | 0.155 | -0.07±0.03 | 0.013 | 3.93±0.43 | <0.001 | 1.00±0.52 | 0.052 |

Abbreviation: PBF, percent body fat, FM, fat mass; FFM, fat free mass; FFM/FM, FFM to FM ratio; ASM, appendicular skeletal muscle mass; ASMI, appendicular skeletal muscle mass index; BMR, basal metabolic rate; VFA, visceral fat area; CHE, cholinesterase; PAB, pre-albumin; TBIL, total bilirubin; DBIL, direct bilirubin; GGT, γ­-glutamyl transferase; AKP, alkaline phosphatase.


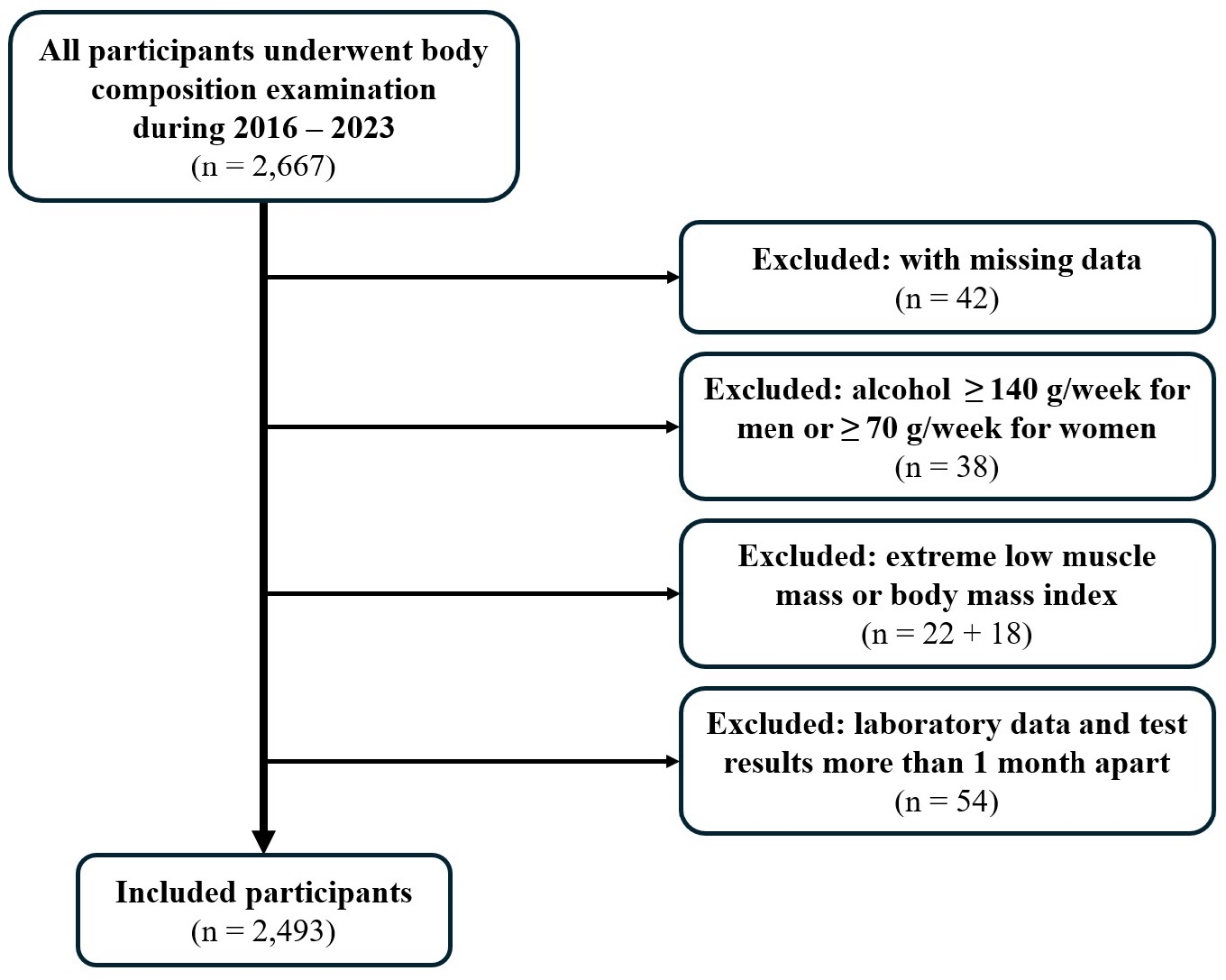


Figure S1. Flowchart of in- and exclusion.
